# Effect of real-time and post-event feedback in out-of-hospital cardiac arrest attended by EMS — A systematic review and meta-analysis

**DOI:** 10.1016/j.resplu.2021.100101

**Published:** 2021-03-12

**Authors:** Rasmus Meyer Lyngby, Mina Nicole Händel, Anne Mielke Christensen, Dimitra Nikoletou, Fredrik Folke, Helle Collatz Christensen, Charlotte Barfod, Tom Quinn

**Affiliations:** a Copenhagen Emergency Medical Services, Telegrafvej 5, 2750 Ballerup, Denmark; b Copenhagen University Hospital Gentofte, Gentofte Hospitalsvej 1, 2900 Hellerup, Denmark; c The Parker Institute, Bispebjerg and Frederiksberg Hospital, Vej 8 11, 2000 Frederiksberg, Denmark; d Kingston University & St George’s, University of London, Cranmer Terrace, Tooting, London SW17 0RE, United Kingdom

**Keywords:** CCD, chest compression depth, CCF, chest compression fraction, CCR, chest compression rate, CI, confidence interval, CINAHL, cumulative index to nursing and allied health literature, CPR, cardiopulmonary resuscitation, EMS, emergency medical service, ERC, European Resuscitation Council, IHCA, in-hospital cardiac arrest, GRADE, grades of recommendation, assessment, development, and evaluation, MD, mean difference, MESH, medical subject headings, OHCA, out-of-hospital cardiac arrest, PICO, population, intervention, comparison and outcome, PRISMA, preferred reporting items for systematic reviews and meta-analyses, ROBINS-I, Cochrane’s risk of bias in non-randomized studies – of interventions, PROSPERO, international prospective register of systematic reviews, RCT, randomised controlled trial, ROSC, return of spontaneous circulation, RR, risk ratio, Out-of-hospital cardiac arrest, Real-time feedback, Post-event feedback, CPR quality

## Abstract

**Objectives:**

A systematic review to determine if cardiopulmonary resuscitation (CPR) guided by either real-time or post-event feedback could improve CPR quality or patient outcome compared to unguided CPR in out-of-hospital cardiac arrest (OHCA).

**Methods:**

Four databases were searched; PubMed, Embase, CINAHL, and Cochrane Library in August 2020 for post 2010 literature on OHCA in adults. Critical outcomes were chest compression depth, rate and fraction. Important outcomes were any return of spontaneous circulation, survival to hospital and survival to discharge.

**Results:**

A total of 9464 studies were identified with 61 eligibility for full text screening. A total of eight studies was included in the meta-analysis. Five studies investigated real-time feedback and three investigated post-event feedback. Meta-analysis revealed that real-time feedback statistically improves compression depth and rate while post-event feedback improved depth and fraction. Feedback did not statistically improve patient outcome but an improvement in absolute numbers revealed a clinical effect of feedback. Heterogenity varied from “might not be important” to “considerable”.

**Conclusion:**

To significantly improve CPR quality real-time and post-event feedback should be combined. Neither real-time nor post event feedback could statistically be associated with patient outcome however, a clinical effect was detected. The conclusions reached were based on few studies of low to very low quality.

**PROSPERO registration:**

CRD42019133881.

## Introduction

Survival from OHCA is highly dependent on cardiopulmonary resuscitation (CPR), which aims to restore and maintain cardio-cerebral perfusion.^1^, ^2^, ^3^ To achieve the best possible blood flow, and thereby outcome, it is important to perform high-quality CPR.^4^

High-quality CPR in adults is defined by the European Resuscitation Council (ERC) as a chest compression rate (CCR) of 100–120 compressions per minute, a chest compression depth (CCD) of 5–6 cm, a chest compression fraction (CCF), which is the percentage of time during a resuscitation event where chest compressions are being performed, of at least 60%, full release of the force exerted to the chest after each compression (recoil), and ventilations with a tidal volume of 500–600 ml and a duration of <1 s.^1^

Out-of-hospital cardiac arrest often occur in suboptimal environments for performing high quality CPR. In addition, paramedics experiences relatively few OHCAs each year which adds to this challenge.^5^ Previous studies have reported that CPR performed by Emergency Medical Services (EMS) does not always reach the recommended standards.^5^, ^6^

To improve CPR various types of CPR feedback have been introduced. Real-time feedback is an audio/visual display of performance metrics to aid paramedics in performing CPR during resuscitation.^6^, ^7^ Post resuscitation debriefing is a retrospective clinical performance evaluation of the team effort following a resuscitation attempt.^8^ The goal of cardiac arrest feedback is to bring resuscitation performance closer to guideline recommendations and ultimately improve patient outcome.^8^

A recent systematic review by Wang et al. identified 11 studies (OHCA and in-hospital cardiac arrest (IHCA)) studies and focused on real-time feedback devices and short term survival.^9^ A similar paper published in 2014 by Kirkbright et al. identified 20 studies (Simulation, OHCA and IHCA) focused on real-time feedback and performance metrics.^10^ An earlier systematic review by Yeung et al. found 28 studies and included both simulation and clinical studies.^11^ Wang et al. concluded that the effectiveness of real-time feedback on short-term survival depended on the type of device used whereas Yeung et al. and Kirkbright et al. concluded that the use of CPR feedback may improve CPR and bring performance closer to guideline recommendations. The combination of simulation, OHCA and IHCA in the previous reviews leaves the effect of feedback solely for OHCA unclear.

The aim of this systematic review was to identify, assess, and synthesise literature published from 2010 (chosen due to guideline changes for CCD, CCR and development in feedback technology) on the use of real-time and post-event feedback for OHCA in a clinical setting. The objectives were to assess the quality of the evidence-base on unguided CPR delivered by emergency medical services during OHCA compared to feedback guided CPR. Furthermore, to assess association to outcome comparing unguided CPR to CPR guided by feedback.

## Methods

We conducted this systematic review and meta-analysis according to the Preferred Reporting Items for Systematic Reviews and Meta-Analyses (PRISMA) guidelines^12^ and based on the principles described in the Grades of Recommendation, Assessment, Development, and Evaluation (GRADE) approach.^13^ The systematic review is structured in accordance to the Population, Intervention, Comparison and Outcome (PICO) framework.^14^

## Protocol and registration

The protocol for this study was registered on International Prospective Register of Systematic Reviews (PROSPERO), registration number: CRD42019133881 and can be accessed on https://www.crd.york.ac.uk/prospero/.

Protocol was submitted on 30 April 2019 and approved on 13 June 2019.

## Eligibility criteria

### Participants

We included primary studies that examined the effect of CPR performed by pre-hospital providers (paramedics, emergency medical technicians, doctors or nurses working in the pre-hospital setting) in adult OHCA cases.

### Interventions and comparison

We included studies that measured CPR quality using defibrillator recording of thoracic movement on the patient during CPR. The defibrillator should be able to quantify frequency of compressions as a minimum. The intervention could be either display of real-time performance metrics during resuscitation or the use of recorded data/non-technical skills evaluation for immediate or delayed feedback after the resuscitation attempt was completed. The intervention(s) (real-time and/or post-event feedback/debriefing) were compared to the group where no real-time feedback was available or post-event feedback/debriefing was not conducted.

### Outcomes

The critical outcomes were improvement in CPR quality comprising of one or more of the following variables; CCD, CCR, recoil, CCF and ventilations. The important outcomes were any return of spontaneous circulation, 30-day survival and survival to discharge.

### Types of studies

Published studies of non-randomised and randomised clinical trials (RCT) as well as before/after observational studies were included. Only studies comparing data from OHCA in adult patients who received manual CPR from emergency medical services were included. Studies had to be published in English or Danish and published from 2010 or later. Editorials, opinion papers, newspaper articles, and other forms of popular media were excluded.

## Information sources

### Search strategy

The overall search strategy comprised of three different approaches; electronic database search, consultation of experts, and a reference search (snowball search) of included studies to identify any additional papers not revealed during the initial database search. Three researchers were involved in the search, screening and data extraction (RML, AMC, MNH). The search was developed by RML in collaboration with a professional research librarian. The following four databases were searched by RML: PubMed, Embase, Cumulative Index to Nursing and Allied Health Literature (CINAHL), and Cochrane Library. The search strategy was developed using medical subject heading terms (MeSH) or equivalent and text words related to our eligibility criteria, i.e., out-of-hospital cardiac arrest, emergency medical services, feedback, audio-visual, real-time, post-event, cardiopulmonary resuscitation, return of spontaneous circulation, survival, neurologic outcome, quality improvement.

The PubMed search can be found in Appendix 1.

## Study selection

The eligibility criteria were applied to the search results, and studies were screened using a three-stage approach to review the title, abstract, and full text.

Studies identified in the searches were imported to Zotero reference manager (version 5.0.82) (Roy Rosenzweig Centre for History and New Media, Virginia, USA) and thereafter exported to Covidence literature screening software (Veritas Health Innovation, Melbourne, Australia), for removal of duplicates and subsequent screening. Screening by title and abstract was conducted independently by two reviewers (RML and AMC). Studies that appeared to meet the inclusion criteria were selected for full text review and subsequently independently screened for eligibility by the same reviewers. The included studies were subject to a reference search and any studies identified through this search were subject to screening of abstract and full text. Discrepancies were resolved through discussion. To test for interrater reliability Cohen’s kappa score was calculated using statistical software SAS 9.4.

## Data extraction

### Data collection

Relevant information from the included studies was extracted using a predefined data extraction template developed for this systematic review. Data were extracted by two reviewers (RML and MNH) using Covidence (Veritas Health Innovation, Melbourne, Australia). Inconsistencies were automatically detected by the software and discrepancies were resolved by discussion and consensus.

### Data items

(1) characteristics of participants (age, gender),
(2) characteristics of cardiac arrest (location, bystander CPR),
(3) settings (type of defibrillator, providers, guidelines, country),
(4) study (design, data collection period, number of included participants, type of intervention).

## Risk of bias and analysis

### Assessment of risk of bias in individual studies

Two authors (RML and MNH) assessed the risk of bias in the included studies. For randomised controlled trials the Cochrane’s risk of bias tool was used.^14^ For non-randomised trials Cochrane’s Risk Of Bias In Non-randomized Studies – of Interventions (ROBINS-I) tool was used.^15^

The Cochrane’s risk of bias tool provided risk assessment for five different domains: Randomization sequence generation; Treatment allocation concealment; Blinding of patients and personnel; Blinding of outcome assessors; Completeness of outcome data; Selective outcome reporting; Other sources of bias. Studies were assessed as either low or high risk of bias within each domain. If nothing was stated in the study, a rating of unclear risk of bias was given.

ROBINS-I provided risk assessment across seven domains; judgement of risk of bias that may arise due to confounding (the pre-specified confounders that the studies were evaluated according to were: sample age and gender, Initial rhythm, bystander CPR, algorithms changing American Heart Association/ERC guidelines), selection of participants, measurement of intervention, departures from intended interventions, missing data, measurement of outcomes and selection of reported results. The studies were assessed as either low, moderate, serious, or critical risk of bias within each domain, and the highest risk of bias judgement, was indicative of the overall judgement. If nothing was stated in the study, a rating of unclear risk of bias was given.

Assessments were done by one reviewer (RML), afterwards the judgement was discussed and reviewed with the other reviewer (MNH).

### Statistical analysis

To test for interrater reliability at full text level Cohen’s kappa score calculations were applied and interpreted as either no agreement (≤0), none to slight agreement (0.01–0.20), fair (0.21–0.40), moderate (0.41–0.60), substantial (0.61–0.80) or as almost perfect agreement (0.81–1.00).^16^

Random effect model was applied for all meta-analyses, with results for real-time feedback and post-event feedback. For dichotomous outcomes, we calculated risk ratio (RR) with corresponding 95% confidence intervals (CI). The effect size of continuous outcomes was assessed as mean difference (MD) and 95% CIs. If applicable, statistical heterogeneity was calculated using I^2^ statistics. The analyses and forest plots were produced in Review Manager Software (version 5.2) (The Nordic Cochrane Centre, The Cochrane Collaboration, Copenhagen, Denmark). Due to the limited number of studies, risk of publication bias could not be estimated.

To assess the risk of inconsistency across studies the Cochrane interpretation of the I^2^ test was used categorising the results as; (1) might not be important risk of heterogeneity (0%–40%), (2) may represent moderate heterogeneity (30%–60%), (3) may represent substantial heterogeneity (50%–90%), (4) considerable heterogeneity (75%–100%). We followed the rough guide of thresholds for the interpretation of I^2^ recommended by Cochrane allowing for 0%–60% heterogeneity.^14^ A sensitivity analysis was applied in estimates revealing heterogeneity >60% and studies driving the heterogeneity was removed to lower the estimates.

### Quality of evidence across studies

To assess the quality of the evidence the GRADE approach was applied. Overall, the ratings of GRADE are as follows: very low, low, moderate, and high certainty in the estimates, which is an indication of the robustness in the interpretations of the results and whether the overall conclusions are likely to change with the inclusion of new studies. For RCT’s the certainty in the estimates starts at high and are assessed for possible downgrading, based on the following domains: overall risk of bias; inconsistency; indirectness; imprecision and publication bias. For observational studies the starting point is low and are assessed for upgrading if the effect size is substantial and there are no issues with confounding, as well as downgrading based on the previous mentioned domains.^13^

The overall quality of evidence was subsequently based upon the lowest quality of the critical outcomes in accordance to the GRADE approach.

## Results

### Study selection

**Fig. 1:**
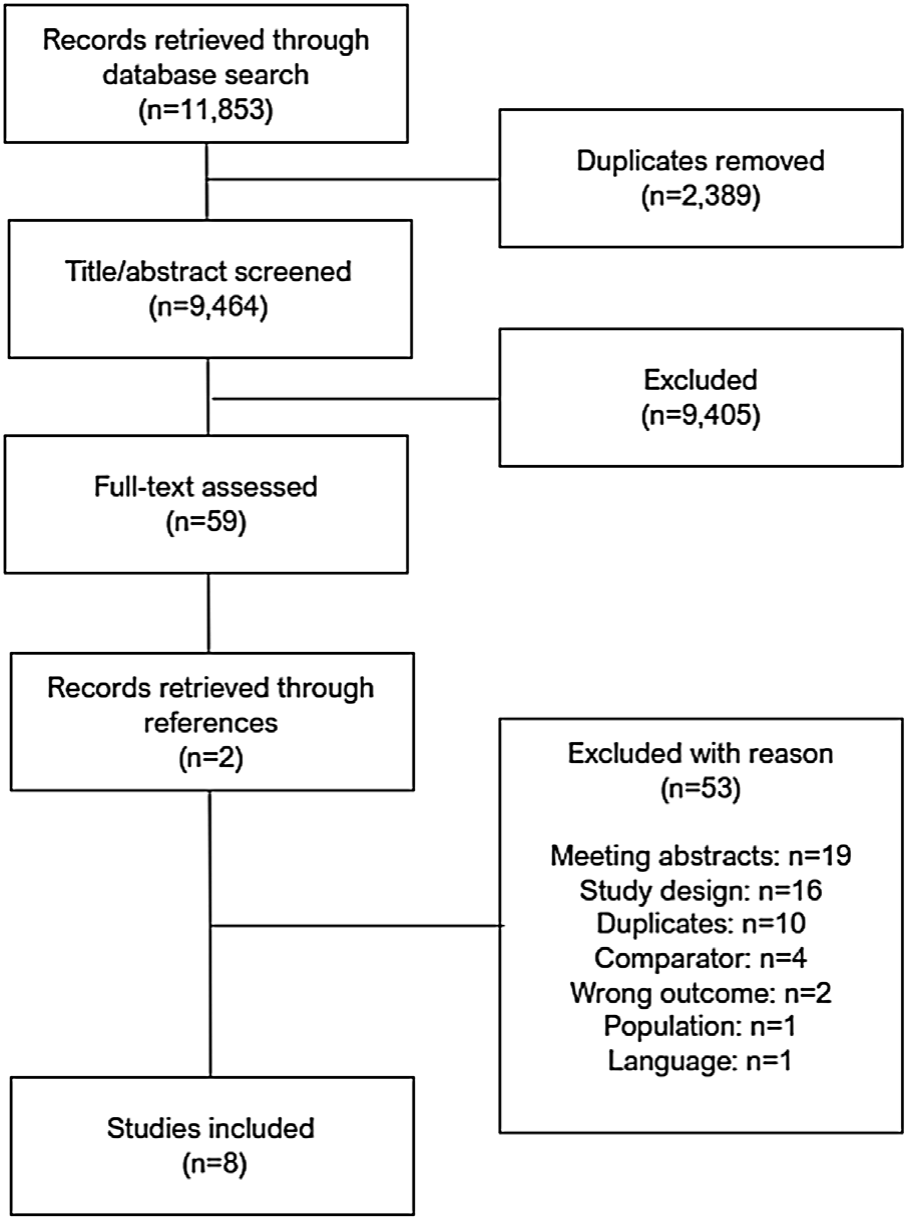
PRISMA flowchart.

A total of 11,853 papers were identified through the electronic database search. A total of 2389 duplicates were removed, leaving 9464 papers for title and abstract screening. Of these, 9405 were excluded. It was not possible to calculate a Cohen’s kappa agreement score at this level, but a proportional agreement of 98.8% was calculated based on 109 conflicts resolved by discussion. There were 59 papers left for full-text screening Reference search and expert network consultation identified 2 additional studies. Of the 61 studies 53 were excluded during full-text screening. Cohen’s kappa agreement score was calculated with a result of 0.87 which equals a ‘strong’ level of agreement^16^ and which was regarded as satisfactory by the team. The total number of papers for extraction of evidence was eight (Fig. 1).^3^, ^17^, ^18^, ^19^, ^20^, ^21^, ^22^, ^23^

### Excluded studies

Of the 53 excluded papers, 19 were abstracts, 16 were excluded due to study design, 10 duplicates were not found by Covidence, 4 excluded for comparator other than real-time or post-event feedback, 2 for wrong outcome, 1 for wrong study population, and 1 for language other than Danish or English (German) (Appendix 2).

### Characteristics of included studies

One study was conducted as a cluster RCT.^19^ All included studies were published in English between 2011 and 2020. Five studies investigated the use of real-time feedback^18^, ^19^, ^20^, ^21^, ^23^ and three investigated the use of post-event debriefing.^3^, ^17^, ^22^ The total number of patients were 4601 ranging from 52 to 1586 in the individual studies. The studies were conducted in the US (n = 3)^18^, ^19^, ^22^ or in Europe (Finland (n = 1),^21^ Spain (n = 1),^20^ Scotland (n = 1),^3^ Holland (n = 1),^17^ Germany (n = 1).^23^ Four studies were conducted according to 2005 resuscitation guidelines,^3^, ^19^, ^20^, ^21^ three according to 2010 guidelines^17^, ^18^, ^22^ and one according to 2015 guidelines.^23^ Of the five studies investigating real-time feedback, three studies^18^, ^19^, ^23^ reported on both CPR performance and patient related outcomes while two studies^20^, ^21^ reported only on patient related outcome. Of the three studies investigating post-event debriefing two^17^, ^22^ reported on CPR performance while one study^3^ reported on CPR performance and patient related outcomes. All post-event feedback studies used a delayed debriefing approach (24–72 h). No information on structure or framework used for debriefing was reported nor was any information on content of the actual feedback session.

The summary of characteristics and reported CPR performance of included studies can be seen in Table 1.

**Table 1:** Characteristics of included studies.

Study and country	Design	Intervention	N	Age (mean)	Gender - Males	Location of arrest (Public)	Bystander CPR	Study period	Device/Guidelines/Providers	Compression depth (cm)	Compression rate (compressions per min)	Compression fraction (%)
Hostler et al.^19^U SA	Cluster randomised controlled trial	Real-time feedback	Control *n* = 771 Intervention *n* = 815 Total *N* = 1586	Control: 66 ± 17 Intervention: 65 ± 17	Control: 62% Intervention: 64%	Control: 13% Intervention: 14%	Control: 50% Intervention: 52%	25 months	Phillips MRx 2005 guidelines (Presumed) EMS providers	Control: 3.78 Intervention: 3.96	Control: 108 Intervention: 103.1	Control: 64 Intervention: 65.9
Bobrow et al.^18^U SA	Cohort Before/after	Real-time feedback	Control *n* = 232 Intervention *n* = 252 Total *N* = 484	Control: 69 (59–79) Intervention: 68 (55–79)	Control: 64.2% Intervention: 68.7%	Control: 14.2% Intervention: 11.9%	Control: 44% Intervention: 35.7%	34 months	Zoll E-series Unclear guidelines 2005 guidelines due to data collection period 2010 guidelines due to reported data Paramedic/EMT	Control: 4.4 Intervention: 5.4	Control: 126 Intervention:105	Control: 65.6 Intervention: 87.3
Sainio et al.^21^F inland	Cohort	Real-time feedback	Total N = 52	Control: 60 ± 20 Intervention: 66 ± 17	Control: 72% Intervention: 67%	Control: 25% Intervention: 40%	No data	18 months	Heartstart MRx 2005 guidelines (Reported) Physicians/paramedics	Control: No data Intervention: No data	Control: No data Intervention: No data	Control: No data Intervention: No data
Leis et al.^20^S pain	Prospective Cohort	Real-time feedback	Control *n* = 784 Intervention *n* = 108 Total *N* = 892	Control: 62.7 ± 18.9 Intervention: 62.6 ± 17.4	Control: 66.2% Intervention:72.2%	No data	No data	37 months	Device not reported 2005 guidelines (Reported) EMS teams	Control: No data Intervention: No data	Control: No data Intervention: No data	Control: No data Intervention: No data
Lakomek et al.^23^*(* *Updated tables)* Germany	Prospective Cohort	Real-time feedback	Control^a^*n* = 95Control b*n* = 94 Intervention *n* = 103 Total *N* = 292	Control^a^*n*: 69.6 ± 14.2Control b*n*: 69.8 ± 16.0 Intervention *n*:71.0 ± 13.0	Control^a^*n*: 64.0% Control^b^*n*: 56.0% Intervention *n*: 70.0%	No data	Control^a^*n*: 41% Control b*n*: 50% Intervention *n*: 55%	25 months	Corpuls with CorPatch 2015 guidelines (Presumed) Physician based EMS system	Control^a^: no dataControl b: 5.25 Intervention: 5.57	Control^a^: 127.81Control b: 122.96 Intervention: 119.15	Control^a^: 80.10Control b: 87.49 Intervention: 88.85
Bleijenberg et al.^17^Hol land	Cohort Before/after	Post-event feedback Debriefing, delayed (presumed), oral, subjective and objective, performance data	Control *n* = 55 Intervention *n* = 69 Total *N* = 124	Control: 68 ± 17 Intervention: 66 ± 17	Control: 71% Intervention: 70%	No data	No data	31 months	LIFEPAK 12 2010 guidelines (presumed) Paramedics and drivers	Control: No data Intervention: No data	Control: No data Intervention: No data	Control: 79 Intervention: 86
Lyon et al.^3^ Scotland	CohortBefore/after	Post-event feedback Debriefing, delayed, written, objective and subjective, performance data	Control *n* = 34 Intervention *n* = 77 Total *N* = 111	Control: 67 ± 17 Intervention: 64 ± 17	Control: 41.2% Intervention: 66.2%	No data	No data	13 months	LIFEPAK 12 2005 guidelines (presumed) Ambulance crews	Control: No data Intervention: No data	Control: 124.5 Intervention: 121.3	Control: 73 Intervention: 79.3
Weston et al.^22^U SA	Cohort Before/after	Post-event feedback Self-assessment form, delayed (72 h), written, objective, performance data	Control *n* = 439 Intervention *n* = 621 Total *N* = 1060	Control: 61.3 ± 17.25 Intervention: 61.4 ± 17	Control: 61.8% Intervention: 58.3%	No data	No data	18 months	ZOLL X-series 2010 guidelines (presumed) BLS and ALS providers	Control: 5 Intervention: 5.5	Control: 109.6 Intervention: 114.8	Control: 79.2 Intervention: 86.4

a No feedback sensor was attached to the patient during resuscitation.

b Feedback sensor was attached to the patient during resuscitation.

## Results of meta-analysis

### Chest compression depth

For CCD we identified low quality of evidence (downgraded for inconsistency of results and imprecision) from one cluster RCT^19^ and very low quality of evidence (downgraded for limitations in design) from three observational studies^18^, ^22^, ^23^ representing 3327 patients. Real-time feedback analysis included three studies^18^, ^19^, ^23^ and post-event feedback one study.^22^

**Fig. 2:**
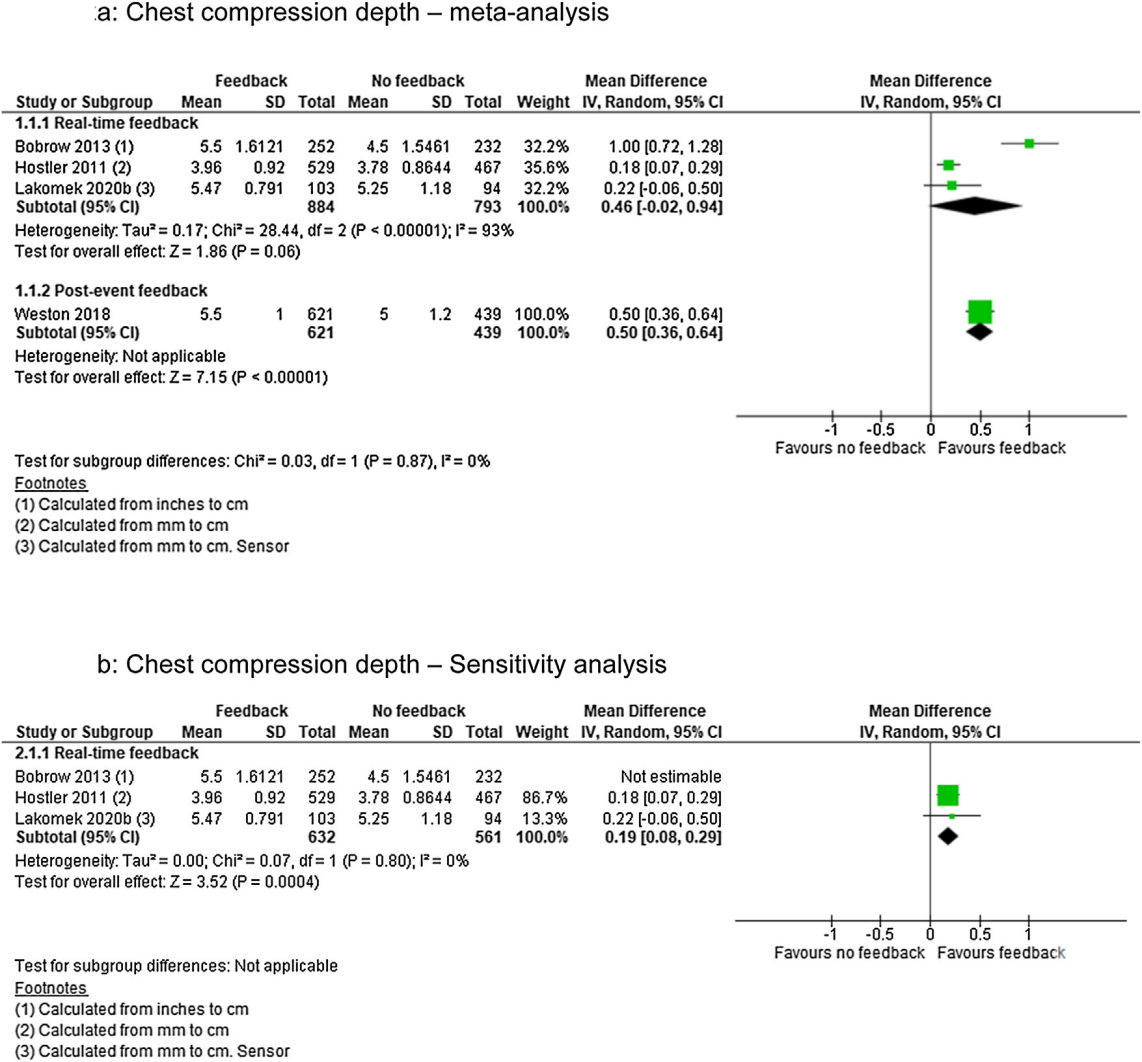
Chest compression depth — meta and sensitivity analysis.

Real-time feedback analysis revealed no significant effect of the intervention (MD 0.46; 95% CI, −0.02, 0.94) but also showed considerable heterogenity (I^2^ = 93%) (Fig. 2a). Removing Bobrow et al.^18^ lowered heterogenity (I^2^ = 0%) and thus changed the effect to favour the intervention (MD 0.19; 95% CI, 0.08, 0.29) (Fig. 2b). For post-event feedback the analysis also favoured the intervention (MD 0.50; 95% CI, 0.36, 0.64) (Fig. 2a).

### Chest compression rate

For CCR we identified low quality of evidence (downgraded for inconsistency of results and imprecision) from one RCT^19^ and very low quality of evidence (downgraded for limitations in design) from four observational studies^3^, ^18^, ^22^, ^23^). Three studies were in the real-time feedback group^18^, ^19^, ^23^ and two were in the post-event feedback group,^3^, ^22^ representing a total of 3533 patients.

**Fig. 3:**
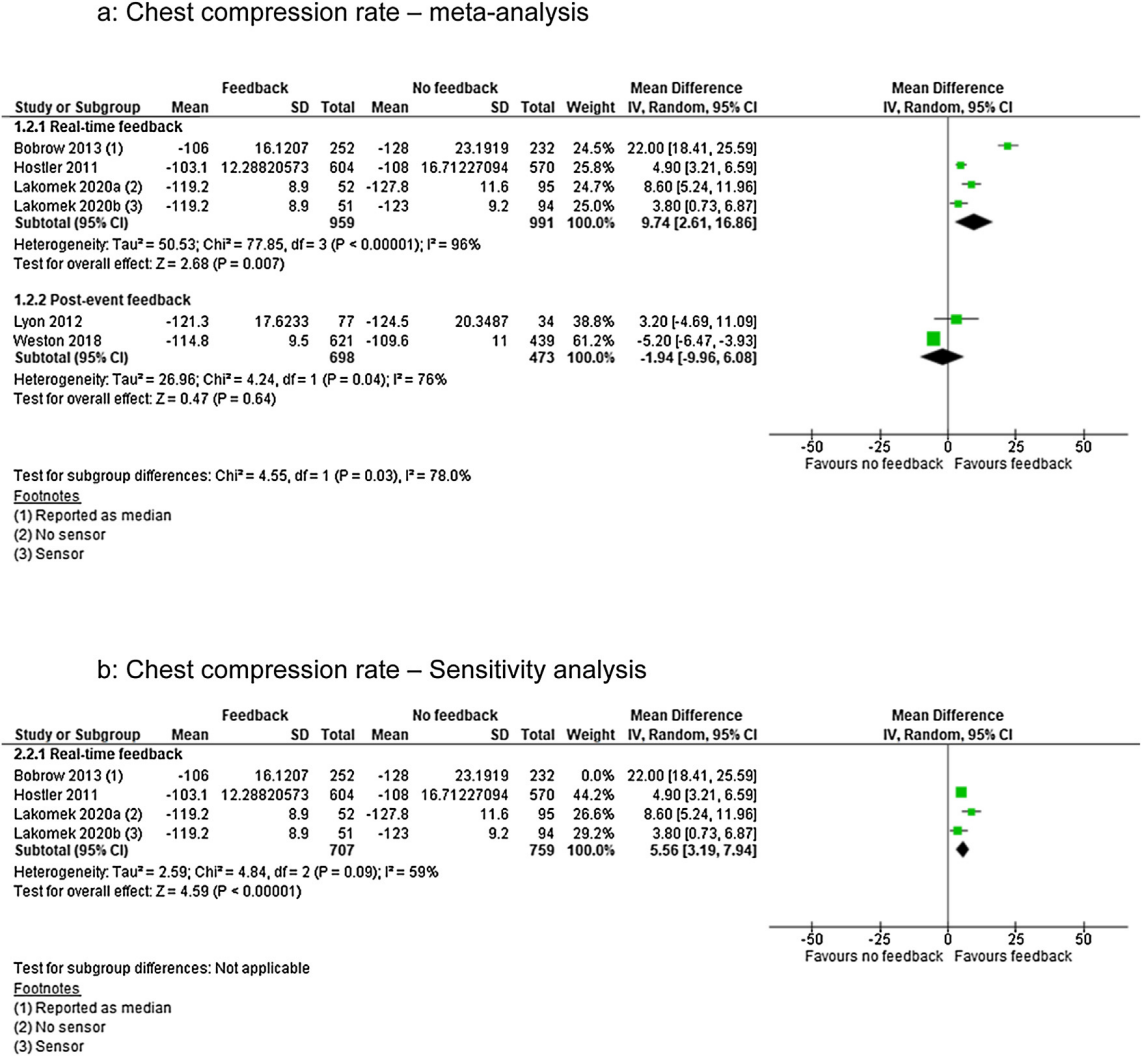
Chest compression rate — meta and sensitivity analysis.

Real-time feedback analysis showed significant effect of feedback (MD 9.74; 95% CI, 2.61, 16.86) but also showed considerable heterogenity (I^2^ = 96%) (Fig. 3a). Removing Bobrow et al.^18^ lowered heterogenity (I^2^ = 59%) but the effect estimates remained in favour of the intervention (MD 5.56; 95% CI, 3.19, 7.94) (Fig. 3b). Post-event feedback analysis showed no significant effect (MD −1.94; 95% CI, −9.96, 6.08) (Fig. 3a) but with considerable heterogenity (I^2^ = 76%). With only two studies^3^, ^22^ no sensitivity analysis was conducted.

### Chest compression fraction

For CCF we identified low quality of evidence from one RCT^19^ (downgraded for inconsistency of results and imprecision) and very low quality evidence from five observational studies^3^, ^17^, ^18^, ^22^^,^^23^ (downgraded for limitations in design). Three studies were in the real-time feedback group^18^, ^19^, ^23^ and three studies were in the post-event feedback^3^, ^17^, ^22^ representing 3,657 patients.

**Fig. 4:**
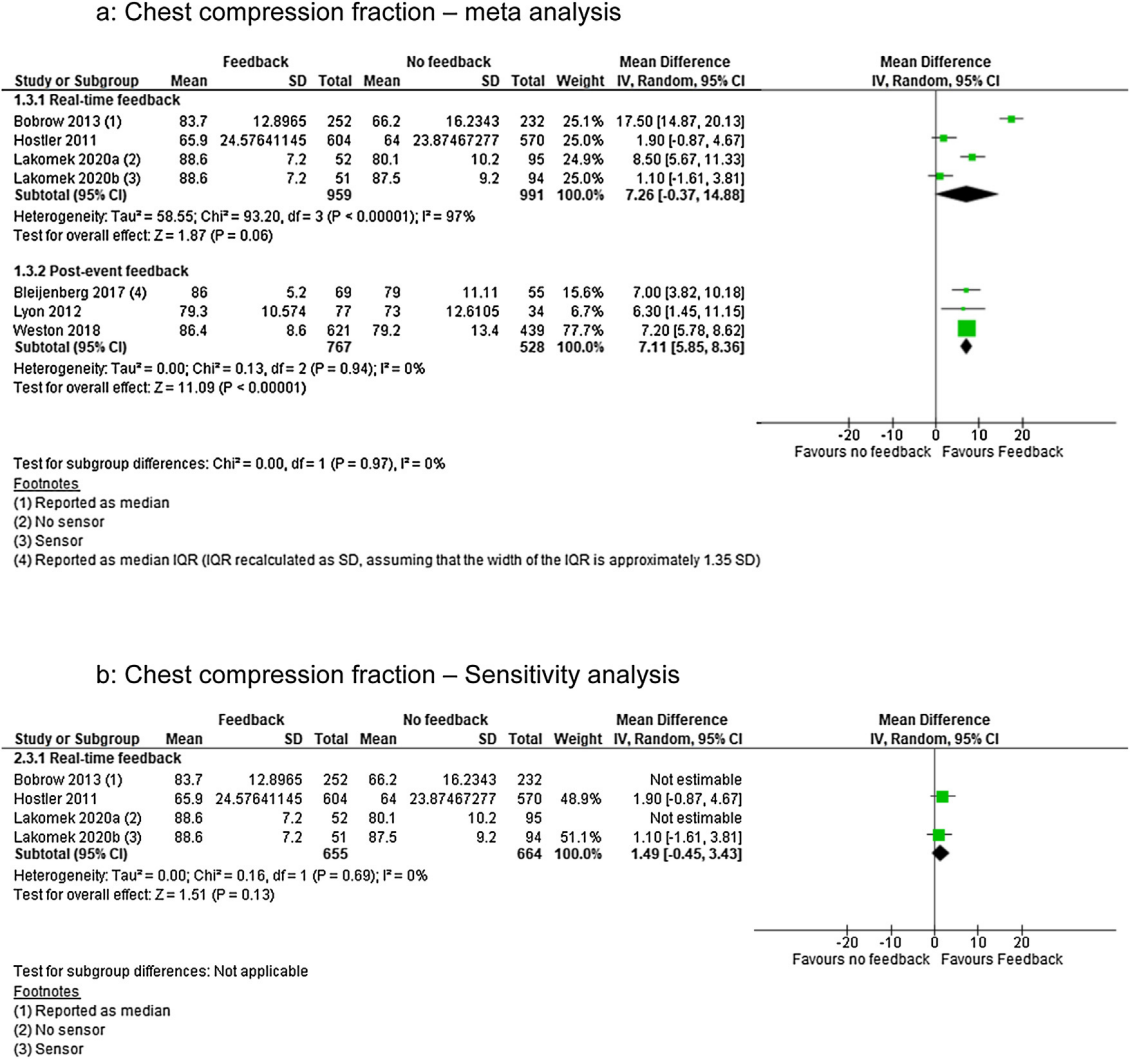
Chest compression fraction — meta and sensitivity analysis.

Real-time feedback analysis showed no significant effect of the intervention (MD 7.26; 95% CI, −0.37, 14.88) but with considerable heterogenity (I^2^ = 97%) (Fig. 4a). Removing Bobrow et al.^18^ and Lakomek et al.^23^ (group a – no sensor) lowered heterogenity (I^2^ = 0%) but did not change the intervention effect (MD 1.49; 95% CI, −0.45, 3.43) (Fig. 4b). Post-event feedback analysis showed significant effect favouring feedback (MD 7.11; 95% CI, 5.85, 8.36) (I^2^ = 0%) (Fig. 4a).

### Return of spontaneous circulation

For ROSC we identified low quality evidence (downgraded for inconsistency of results and imprecision) from one cluster RCT^19^ and very low quality evidence (downgraded for limitations in study design) from five observational studies.^3^, ^18^, ^20^, ^21^^,^^23^ Five studies in the real-time feedback group^18^, ^19^, ^20^, ^21^, ^23^ and one in the post-event feedback group^3^ represented a total of 3,417 patients.

**Fig. 5:**
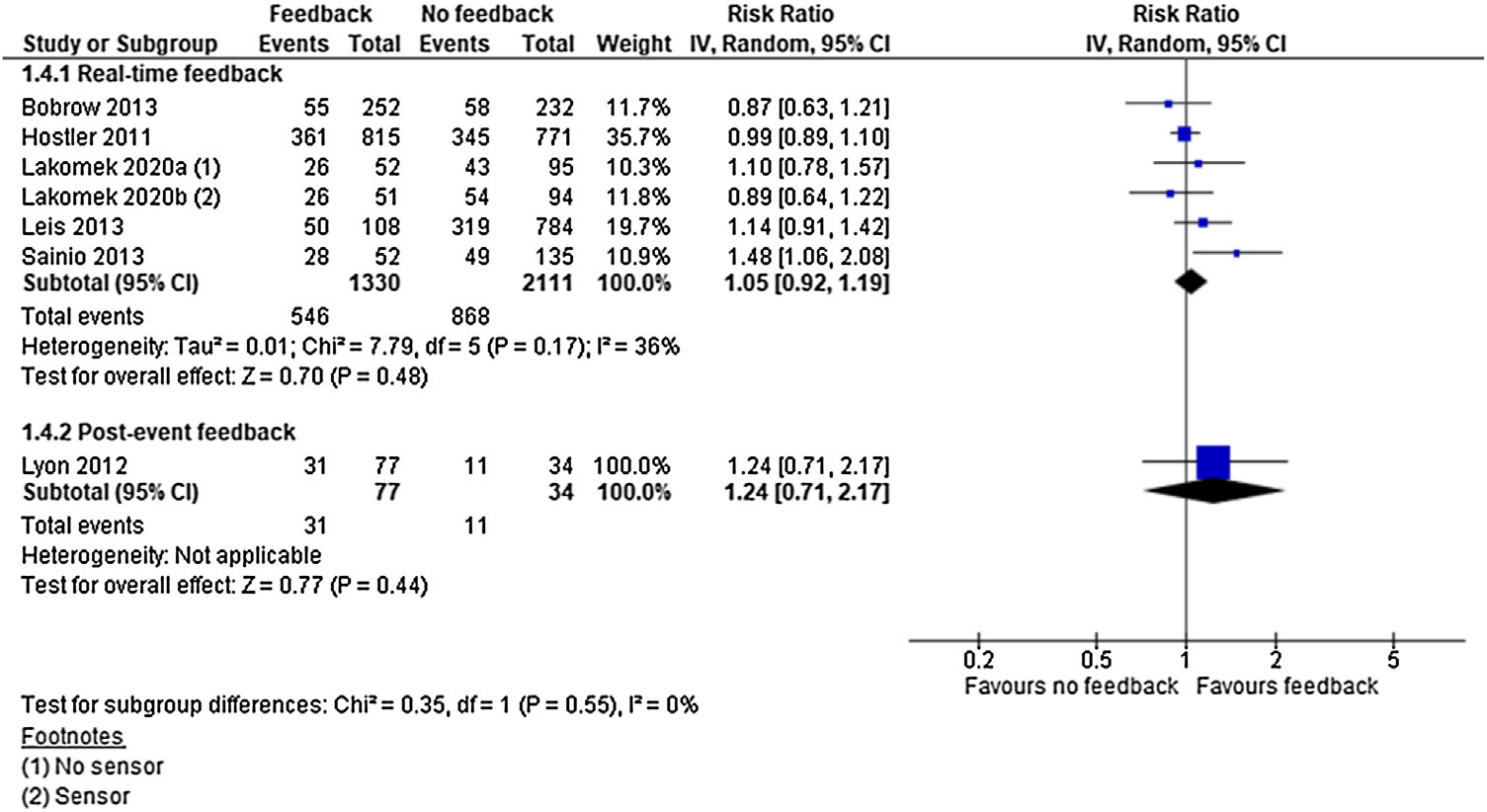
Return of spontaneous circulation — meta-analysis.

Neither real-time feedback (RR 1.05; 95% CI, 0.92, 1.19) (I^2^ = 36%) nor post-event feedback (RR 1.24; 95% CI, 0.71, 2.17) showed a significant effect on ROSC (Fig. 5). In absolute numbers the clinical effect of feedback versus no feedback was 5.4 individuals more per 1000 that regained ROSC with the intervention.

### Survival to hospital/sustained return of spontaneous circulation

For survival to hospital/sustained ROSC we identified low quality evidence (downgraded for inconsistency of results and imprecision) from one cluster RCT^19^ and very low quality evidence (downgraded for limitations in study design or execution) from two observational studies^21^, ^23^ representing 1930 patients.

**Fig. 6:**
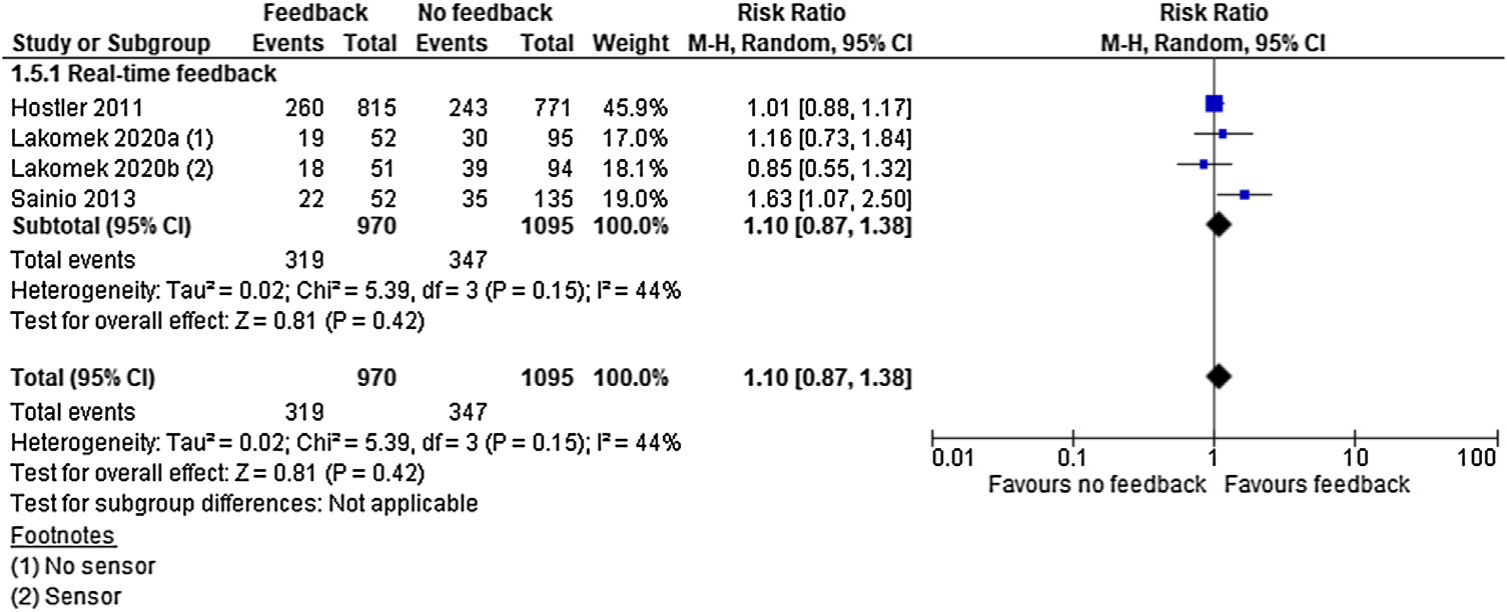
Survival to hospital — meta-analysis.

All studies intervened by real-time feedback and analysis revealed no significant effect of the intervention (RR 1.10; 95% CI, 0.87, 1.38) (I^2^ = 44%) (Fig. 6). In absolute numbers the clinical effect of feedback versus no feedback was 12 individuals more per 1000 that are surviving to hospital with intervention.

### Survival to hospital discharge

For survival to hospital discharge we identified low quality evidence (downgraded for inconsistency of results and imprecision) from one cluster RCT^19^ and very low quality evidence (downgraded for limitations in study design) from three observational studies.^3^, ^17^, ^18^ Two studies^18^, ^19^ were included in the real-time feedback group and two^3^, ^17^ in the post-event feedback group, representing a total of 2305 patients.

**Fig. 7:**
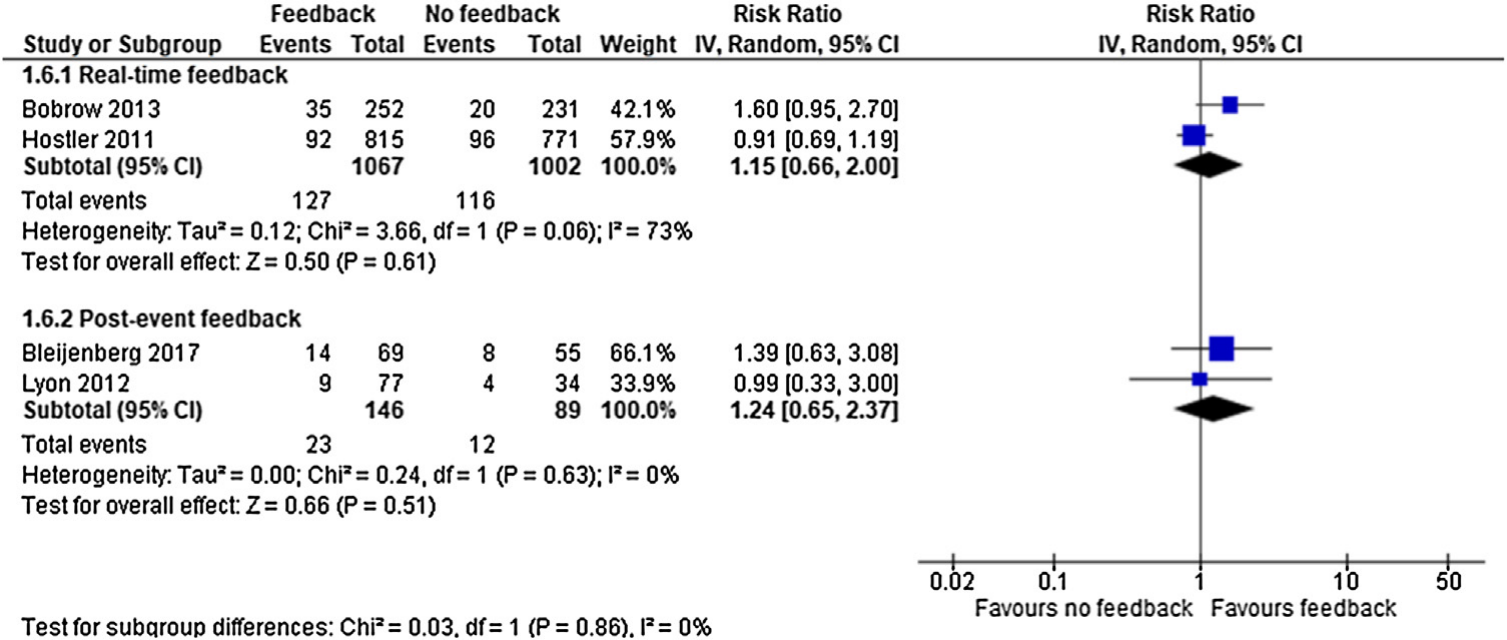
Survival to hospital discharge — meta-analysis.

Real-time feedback analysis revealed no significant effect of the intervention (RR 1.15; 95% CI, 0.66, 2.00) but showed substantial heterogeneity (I^2^ = 73%). With only two studies^18^, ^19^ no sensitivity analysis was conducted. Post-event feedback analysis revealed no significant effect of the intervention (RR 1.24; 95% CI, 0.65, 2.37) (I^2^ = 0%) (Fig. 7). In absolute numbers the clinical effect of feedback versus no feedback was 6.3 individuals more that discharged alive per 1000.

## Risk of bias

### Results from the Cochrane Risk of Bias tool assessment

The assessment showed that sequence generation and allocation concealment were rated unclear, due to insufficient description. Blinding of participants and outcome assessors were rated low. Due to the nature of the studies blinding of participants were not feasible (participants were aware of when they received feedback), but it is unlikely that this will influence the reporting of any of the outcomes of interest. Incomplete outcome data was rated as high due to insufficient information on the handling of missing data. Selective outcome reporting and other sources of bias were rated low.

### Results from ROBINS-I tool assessment

The assessment of the observational studies showed that within domain 1 (confounding) five studies were rated as critical.^3^, ^17^, ^20^, ^21^^,^^23^ Within domain 2 (selection of participants) two were rated as serious.^20^, ^21^ Within domain 4 (departure from interventions) one was rated as serious^21^ and within domain 5 (missing data) one was rated serious.^23^ The remaining studies were all rated as moderate or low within the domains and can be seen in Table 2.

**Table 2:** Risk of bias.

Observational studies – ROBINS - I								
Study	Domain 1Confounding	Domain 2Selection of participants	Domain 3Classification of interventions	Domain 4Departure from intended interventions	Domain 5Missing data	Domain 6Measurements of outcome	Domain 7Selection of reported results	Overall^a^
Bleijenberg^17^	Critical	Moderate	Moderate	Low	Moderate	Low	Low	Critical
Bobrow^18^	Moderate	Moderate	Low	Moderate	Low	Low	Low	Moderate
Leis^20^	Critical	Serious	Low	Low	Moderate	Low	Low	Critical
Lyon^3^	Critical	Moderate	Low	Low	Moderate	Low	Low	Critical
Lakomek^23^	Critical	Moderate	Low	Low	Serious	Low	Low	Critical
Sainio^21^	Critical	Serious	Moderate	Serious	Moderate	Moderate	Low	Critical
Weston^22^	Moderate	Moderate	Low	Low	Moderate	Low	Low	Moderate

Randomised controlled trials – Cochranes Risk of Bias tool							
Study	Sequence Generation	Allocation concealment	Blinding of participants	Blinding of outcome assessors	Incomplete outcome data	Selective outcome reporting	Other sources of bias
Hostler^19^	Unclear	Unclear	Low	Low	High	Low	Low

a Highest risk of bias judgement, was indicative of the overall judgement.

### Results from GRADE assessment

Subsequent rating of the overall certainty of effect estimates was low for all outcomes due to serious inconsistency of the results and imprecision. For the observational studies the overall certainty of the evidence was very low, due to serious risk of bias, in addition to the inconsistency of the results and imprecision.

## Discussion

### Summary of evidence

We conducted a systematic review on the use of feedback exclusively for OHCA in the clinical setting. Real-time and post event feedback differ in timing and thus ability to affect outcome. Real-time feedback allows for in-situ change where post-event feedback can only affect subsequent similar events. When adjusting for inconsistencies on CCD, CCR and CCF we found real-time feedback to improve CCD and CCR, while CCF remained insignificant. Post-event feedback improved CCD and CCF (no inconsistencies) but not CCR (unable to adjust). Neither real-time nor post event feedback proved superior for ROSC, survival to hospital (no inconsistencies) or survival to discharge (unable to adjust in real-time feedback group). Based on a wide search, our systematic review included studies of low to very low quality of evidence.

Compared to previous work Wang et al. did not report performance metrics and analysed by feedback device. Therefore, comparing our results to those of Wang et al. should be cautioned but indications are that real-time feedback may not affect patient outcome. Compared to the findings of Kirkbright et al. who found no change in patient centred outcome favouring feedback, we found a non-significant result favouring feedback.

### Effect of feedback on CPR quality metrics

The effect of feedback on CPR quality varies depending on type of feedback and component (CCD, CCR or CCF). Several reasons can explain these finding and their partial lack in confirming the hypothesis of this systematic review. One could be the “one size fits all” approach to CPR, where guideline recommendations are made based on the average size adult. Obese or underweight patients may require paramedics to adjust their compression depth which can result in poor guideline compliance, but still be a clinically correct compression performance. Similar effect may be caused by patients receiving chest compressions on a soft surface (mattress effect).^24^, ^25^

### Effect of feedback on patient outcome

Our synthesis found that feedback was not associated with improved patient outcome. Several reasons may contribute to the explanation of this. A statistical improvement in CPR quality may be marginal and therefore not enough to provide a clinical detectible improvement but more importantly, CPR quality consists of five independent, yet interlinked, variables. An improvement in one of five quality variables cannot reasonably be expected to improve patient outcome. Finally, CPR quality is only one of several steps in survival from OHCA. Time to first shock, bystander CPR and response time are all confounding factors that may also affect patient outcome despite CPR feedback.

### Strengths

The strength of our work, and how it differentiates to previous contributions, is its focus solely on OHCA. Furthermore, we included both types of feedback commonly used for OHCA in our analysis thereby providing a transparent evidence base for feedback in clinical quality improvement and management. This systematic review thereby acknowledged the environmental and resource challenges in the pre-hospital setting during attempted resuscitation and thereby provides an updated summary of evidence regarding use of feedback solely for OHCA.

### Limitations

Feedback for OHCA is sparsely investigated, as reflected in the low number of studies eligible for inclusion, and their quality ratings. The studies included were low to very low-quality evidence, thus our confidence in the estimates reflects this. Removing studies of low quality or high risk of bias was deliberately disregarded in order to do a systematic review in this important, but sparsely investigated field of pre-hospital research. Therefore, the conclusions reached hold a high degree of uncertainty.

Performance feedback is limited in its nature as minor improvements, or adjustments within the high-quality spectra, may not be detectable in patient outcome. As most of the studies included were not powered to address outcome this will only increase the lack of ability to detect patient centred outcome following performance changes. Post-event feedback can be delivered in several ways based on various objective or subjective observations. The results reached is based on delayed feedback and may therefore not be transferable to other types of post-event feedback. Not all studies clearly defined the types of guidelines used during data collection. Therefore, it was not possible to set a guideline determined inclusion. To minimise the risk of papers using pre 2005 guidelines a publication year of 2010 or later was decided.

### Considerations for further research

Further research on the use of OHCA CPR feedback using contemporary guideline recommendations should be considered focusing on both CPR quality and patient outcomes.

## Conclusions

Based on studies of low to very-low quality, real-time feedback improved CCD and CCR while post-event feedback improved CCD and CCF. Neither real-time nor post event feedback improved ROSC, survival to hospital or survival to discharge. High-quality research on feedback approaches is needed.

## Authors’ contribution to the manuscript

Rasmus Meyer Lyngby (RML), Mina Nicole Händel (MNH), Anne Mielke Christensen (AMC), Dimitra Nikoletou (DN), Fredrik Folke (FF), Helle Collatz Christensen (HCC), Charlotte Barfod (CB), Tom Quinn (TQ).

Conception and design of the study: RML

Acquisition of data: RML, MNH, AMC,

Analysis and interpretation of data: RML, MNH

Drafting the article/revising it critically: RML, MNH, AMC, DN, FF, HCC, CB, TQ

Final approval: RML, MNH, AMC, DN, FF, HCC, CB, TQ

## Ethics information

Not applicable. No patient sensitive information in content.

## Conflict of interest

None.

## Funding

This review has been supported by the TrygFoundation. Copenhagen Emergency Medical Services receives funding from 10.13039/501100004102Laerdal Foundation. Parker institute is supported by the 10.13039/100001275Oak Foundation. Funders had no role in study design, data collection, data analysis or data interpretation, writing of the report or to submit the article for publication.
