# Supplementary material for: Effect of real-time and post-event feedback in out-of-hospital cardiac arrest attended by EMS — A systematic review and meta-analysis
Source: Resusc Plus. 2021 Mar 12;6:100101. doi: 10.1016/j.resplu.2021.100101 (PMC8244394; doi:10.1016/j.resplu.2021.100101)
Supplement: Supplementary file 1 [file mmc1.docx]

***Appendix 1 – PubMed search***

Search conducted on 17 august 2020

Filters: Date (1 January 2010 – 17 August 2020)

Hits: 3502

***(("2010/01/01"[Date - Publication] : "2020/08/17"[Date - Publication])) AND (((((((((((((((((((((Out-Of-Hospital Cardiac Arrest*[Text Word]) OR Ambulance technician*[Text Word]) OR Emergency medical services[Text Word]) OR Death, sudden, cardiac[Text Word]) OR Heart arrest*[Text Word]) OR OHCA[Text Word]) OR Cardiac arrest*[Text Word]) OR Cardiopulmonary arrest*[Text Word]) OR Pre-hospital[Text Word]) OR Prehospital[Text Word]) OR Pre hospital[Text Word]) OR EMS[Text Word]) OR Paramedic*[Text Word]) OR Emergency medical technician*[Text Word]) OR EMT[Text Word])) OR ((((Out-Of-Hospital Cardiac Arrest[MeSH Terms]) OR Heart arrest[MeSH Terms]) OR Death, sudden, cardiac[MeSH Terms]) OR Emergency medical services[MeSH Terms]))) AND (((((((((((((Feedback[Text Word]) OR Evaluat*[Text Word]) OR Guid*[Text Word]) OR CPR aid*[Text Word]) OR CPRmeter[Text Word]) OR Audiovisual[Text Word]) OR Post-event[Text Word]) OR Real-time[Text Word]) OR Prompt[Text Word]) OR Assisted CPR[Text Word]) OR Debrief*[Text Word])) OR Feedback[MeSH Terms])) AND (((((((Resuscitation[Text Word]) OR Unguid*[Text Word]) OR Chest compress*[Text Word]) OR CPR[Text Word]) OR Cardiopulmonary resuscitation[Text Word])) OR Resuscitation[MeSH Terms])) AND ((((((((((Quality improvement*[Text Word]) OR Blood circulation[Text Word]) OR Quality indicat*[Text Word]) OR Mortality[Text Word]) OR Survival[Text Word]) OR Return of spontaneous circulation[Text Word]) OR ROSC[Text Word]) OR Neurologic outcome*[Text Word])) OR ((((((Quality improvement[MeSH Terms]) OR Blood circulation[MeSH Terms]) OR Survival[MeSH Terms]) OR Mortality[MeSH Terms]) OR health care[Other Term]) OR Quality indicators[MeSH Terms])))***
