# Supplementary material for: Effect of real-time and post-event feedback in out-of-hospital cardiac arrest attended by EMS — A systematic review and meta-analysis
Source: Resusc Plus. 2021 Mar 12;6:100101. doi: 10.1016/j.resplu.2021.100101 (PMC8244394; doi:10.1016/j.resplu.2021.100101)
Supplement: Supplementary file 2 [file mmc2.docx]

**Appendix 2 – Excluded studies at full text**

[1]

Lyon R, Gowens P, Egan G, Andrews P, Clegg G. 03 Back to basics--ECG impedance analysis for CPR quality control and feedback after out-of-hospital cardiac arrest: a pilot study. Emergency Medicine Journal 2011;28:237–8. <https://doi.org/10.1136/emj.2010.108597.3>.

[2]

Clarke S, Lyon RM, Milligan DJ, Clegg GR. 013 Resuscitation feedback and targeted education improves quality of pre-hospital resuscitation in Scotland. Emergency Medicine Journal 2011;28:A6–A6. <https://doi.org/10.1136/emermed-2011-200617.13>.

[3]

Lyon R, Gowens P, Egan G, Andrews P, Clegg G. 016 Back to basics--ECG impedance analysis for CPR quality control and feedback after out-of-hospital cardiac arrest: a pilot study. Emergency Medicine Journal 2011;28:e1–e1. <https://doi.org/10.1136/emj.2010.108605.16>.

[4]

Lyon R, Gowens P, Egan G, Andrews P, Clegg G. 016 Back to basics--ECG impedance analysis for CPR quality control and feedback after out-of-hospital cardiac arrest: a pilot study. Emergency Medicine Journal 2011;28:e1–e1. <https://doi.org/10.1136/emj.2010.108605.16>.

[5]

Bibbens S, Bernardo E, Delaney H, Matos R. 262: IMPROVING COMPRESSION QUALITY AT A SINGLE INSTITUTION THROUGH REAL-TIME CPR FEEDBACK. Critical Care Medicine 2016;44:142. <https://doi.org/10.1097/01.ccm.0000508942.49804.5b>.

[6]

Brinkrolf P, Lukas R, Harding U, Thies S, Gerss J, Van Aken H, et al. A better understanding of ambulance personnel’s attitude towards real-time resuscitation feedback. International Journal for Quality in Health Care 2018;30:110–7. <https://doi.org/10.1093/intqhc/mzx189>.

[7]

Freese J, Menegus Mark, Slesinger Todd, Silverman Robert, Keller Norma, Dillworth Judy, et al. Abstract 72: Addition of Real-Time CPR Feedback Improves Immediate Outcomes for Out-of-Hospital Cardiac Arrest. Circulation 2014;130:A72–A72. <https://doi.org/10.1161/circ.130.suppl_2.72>.

[8]

Bobrow Bentley J, Spaite Daniel W, Stolz Uwe, Panchal Ashish R, Vadeboncoeur Tyler F, Silver Annemarie, et al. Abstract 208: Achieving the 2010 AHA Guideline Metrics for CPR Quality Is Associated with Improved Survival from Out-of-Hospital Cardiac Arrest. Circulation 2011;124:A208–A208. <https://doi.org/10.1161/circ.124.suppl_21.A208>.

[9]

Vadeboncoeur Tyler, Silver Annemarie, Murphy Ryan Anne, Moon Sungwoo, Tobin John, Venuti Mark, et al. Abstract 306: Use of Real-Time Audiovisual CPR Feedback is Associated With Improved CPR Quality During Patient Transfer From the Scene to the Ambulance in Out-of-Hospital Cardiac Arrest. Circulation 2013;128:A306–A306. <https://doi.org/10.1161/circ.128.suppl_22.A306>.

[10]

Kirkbright S, Finn J, Tohira H, Bremner A, Jacobs I, Celenza A. Audiovisual feedback device use by health care professionals during CPR: A systematic review and meta-analysis of randomised and non-randomised trials. Resuscitation 2014;85:460–71. <https://doi.org/10.1016/j.resuscitation.2013.12.012>.

[11]

Lyon R. Back to basics – ECG impedance analysis for CPR quality control and feedback after out-of-hospital cardiac arrest: a pilot study. Emergency Medicine Journal 2010;27:A6–7. <https://doi.org/10.1136/emj.2010.103150.17>.

[12]

Heradstveit BE, Sunde G-A, Heltne J. Capnography during CPR- Is it useful ? Resuscitation 2012;83:e9. <https://doi.org/10.1016/j.resuscitation.2012.08.023>.

[13]

Burrell L, Rice A. Cerebral oximetry monitoring in OHCA. Journal of Paramedic Practice 2018;10:517–23. <https://doi.org/10.12968/jpar.2018.10.12.517>.

[14]

Lukas R-P, Gräsner JT, Seewald S, Lefering R, Weber TP, Van Aken H, et al. Chest compression quality management and return of spontaneous circulation: A matched-pair registry study. Resuscitation 2012;83:1212–8. <https://doi.org/10.1016/j.resuscitation.2012.03.027>.

[15]

Correction. Annals of Emergency Medicine 2015;65:344. <https://doi.org/10.1016/j.annemergmed.2015.01.019>.

[16]

Kizyma V, Karas J, Ganyovics A, Vitkova M. CPR quality control – The road from training to real performance analysis. Resuscitation 2015;96:68. <https://doi.org/10.1016/j.resuscitation.2015.09.162>.

[17]

Abella BS. CPR-SF Technology Provides Instant Feedback. JEMS 2011. <https://www.jems.com/2011/02/28/cpr-sf-technology-provides-ins/> (accessed March 9, 2020).

[18]

Couper K, Perkins GD. Debriefing after resuscitation: Current Opinion in Critical Care 2013;19:188–94. <https://doi.org/10.1097/MCC.0b013e32835f58aa>.

[19]

Weston BW, Jasti J, Lerner EB, Szabo A, Aufderheide TP, Colella MR. Does an individualized feedback mechanism improve quality of out-of-hospital CPR? Resuscitation 2017;113:96–100. <https://doi.org/10.1016/j.resuscitation.2017.02.004>.

[20]

Hayakawa M. Effect of feedback by NIRO-CCR1 during cardiopulmonary resuscitation for patients with out-of-hospital cardiac arrest 2020. <https://upload.umin.ac.jp/cgi-open-bin/ctr_e/ctr_view.cgi?recptno=R000020975> (accessed March 23, 2020).

[21]

Chandra S. Effect of real-time automated and delayed summative feedback on CPR quality in adult out-of-hospital cardiac arrest: A prospective multicenter controlled clinical trial. Academic Emergency Medicine 2011;18:S4–249. <https://doi.org/10.1111/j.1553-2712.2011.01073.x>.

[22]

Melina MR, Handbury JD, Janney J. Evaluation of ventilatory rates and the benefits of an immediate feedback device with and without supplementary instruction on out-of-hospital resuscitations. Academic Emergency Medicine 2012;19:S4–393. <https://doi.org/10.1111/j.1553-2712.2012.01332.x>.

[23]

Rupp D, Ploeger B, Jerrentrup A, Wranze E, Kunkel R, Kill C. Five years after implementation: structured team-feedback can improve adherence to guidelines and rate of survival after cardiac arrest. Resuscitation 2019;142:e19. <https://doi.org/10.1016/j.resuscitation.2019.06.052>.

[24]

Watford CA. High quality resuscitation bundles include real-time audiovisual CPR feedback. Resuscitation 2015;87:e1. <https://doi.org/10.1016/j.resuscitation.2014.04.035>.

[25]

Nolan JP. High-quality cardiopulmonary resuscitation: Current Opinion in Critical Care 2014;20:227–33. <https://doi.org/10.1097/MCC.0000000000000083>.

[26]

Abella BS. High-quality cardiopulmonary resuscitation: current and future directions. Current Opinion in Critical Care 2016;22:218–24. <https://doi.org/10.1097/MCC.0000000000000296>.

[27]

Leis C, Hernández R, Paterna PC, González V, Ochoa MJ, Torres E. How do you perform CPR? Q-CPR: A system for control CPR. Resuscitation 2010;81:S50. <https://doi.org/10.1016/j.resuscitation.2010.09.210>.

[28]

Hopkins CL, Burk C, Moser S, Meersman J, Baldwin C, Youngquist ST. Implementation of Pit Crew Approach and Cardiopulmonary Resuscitation Metrics for Out‐of‐Hospital Cardiac Arrest Improves Patient Survival and Neurological Outcome. JAHA 2016;5. <https://doi.org/10.1161/JAHA.115.002892>.

[29]

Hubner P, Lobmeyr E, Wallmüller C, Poppe M, Datler P, Keferböck M, et al. Improvements in the quality of advanced life support and patient outcome after implementation of a standardized real-life post-resuscitation feedback system. Resuscitation 2017;120:38–44. <https://doi.org/10.1016/j.resuscitation.2017.08.235>.

[30]

Cheng A, Brown LL, Duff JP, Davidson J, Overly F, Tofil NM, et al. Improving Cardiopulmonary Resuscitation With a CPR Feedback Device and Refresher Simulations (CPR CARES Study): A Randomized Clinical Trial. JAMA Pediatr 2015;169:137. <https://doi.org/10.1001/jamapediatrics.2014.2616>.

[31]

Nassar BS, Kerber R. Improving CPR Performance. Chest 2017;152:1061–9. <https://doi.org/10.1016/j.chest.2017.04.178>.

[32]

Koyama Y, Inoue Y, Hisago S, Marushima A, Hagiya K, Yamasaki Y, et al. Improving the neurological prognosis following OHCA using real-time evaluation of cerebral tissue oxygenation. The American Journal of Emergency Medicine 2018;36:344.e5-344.e7. <https://doi.org/10.1016/j.ajem.2017.11.029>.

[33]

Lyon RM, Clarke S, Gowens P, Egan G, Clegg GR. Improving the quality of pre-hospital resuscitation through defibrillator feedback reporting and CPR training. Resuscitation 2010;81:S10. <https://doi.org/10.1016/j.resuscitation.2010.09.054>.

[34]

Cortegiani A, Russotto V, Baldi E, Contri E, Raineri SM, Giarratano A. Is it time to consider visual feedback systems the gold standard for chest compression skill acquisition? Crit Care 2017;21:166. <https://doi.org/10.1186/s13054-017-1740-z>.

[35]

Salvucci Jr. A. Literature Review: Real-time CPR Feedback and Return of Spontaneous Circulation. EMS World n.d. <https://www.emsworld.com/article/10243975/literature-review-real-time-cpr-feedback-and-return-spontaneous-circulation> (accessed March 9, 2020).

[36]

Wik L. Near-infrared spectroscopy during cardiopulmonary resuscitation and after restoration of spontaneous circulation: a valid technology? Current Opinion in Critical Care 2016;22:191–8. <https://doi.org/10.1097/MCC.0000000000000301>.

[37]

Lakomek F, Brinkrolf P, Lukas R-P, Mennewisch A, Steinsiek N, Gutendorf P, et al. Osnabrück Study on Cardiac Arrest: A prospective trial on the introduction of a real-time feedback system in out-of-hospital resuscitation. Resuscitation 2017;118:e52–3. <https://doi.org/10.1016/j.resuscitation.2017.08.130>.

[38]

Sulzgruber P, Hubner P, Lobmeyr E, Wallmueller C, Poppe M, Datler P, et al. P3021A standardized real-life post-resuscitation feedback-system improves the quality of advanced life support in out-of-hospital cardiac arrest. Eur Heart J 2017;38. <https://doi.org/10.1093/eurheartj/ehx504.P3021>.

[39]

Cheng A, Overly F, Kessler D, Nadkarni VM, Lin Y, Doan Q, et al. Perception of CPR quality: Influence of CPR feedback, Just-in-Time CPR training and provider role. Resuscitation 2015;87:44–50. <https://doi.org/10.1016/j.resuscitation.2014.11.015>.

[40]

Gao M, Liu C, Gehman S, Rea T, Blackwood JE, Studnek JR, et al. Quality Analysis of Cardiopulmonary Resuscitation From Two Emergency Medical Service Systems Using a Feedback Device. Circulation 2019;140:A296–A296.

[41]

Magnet IAM, Nürnberger AN, Warenits AM, Girsa M, Glück H, Maszar H, et al. Quality analysis of cardiopulmonary resuscitation of the ambulance service of Vienna: A preliminary analysis. Resuscitation 2010;81:S12–3. <https://doi.org/10.1016/j.resuscitation.2010.09.062>.

[42]

Bobrow B, Marion L, Heightman A. Quality makes the difference: closing the CPR knowledge-practice gap. FireRescue Magazine n.d.

[43]

Alonso D, Vaqueriza I, Corcuera C, Vicente F, Aramendi E, Irusta U, et al. Quality of chest compressions for EMT CPR in the Basque Autonomous Community. Resuscitation 2015;96:71–2. <https://doi.org/10.1016/j.resuscitation.2015.09.169>.

[44]

Gruber J, Stumpf D, Zapletal B, Neuhold S, Fischer H. Real-time feedback systems in CPR. Trends in Anaesthesia and Critical Care 2012;2:287–94. <https://doi.org/10.1016/j.tacc.2012.09.004>.

[45]

Weston BW, Jasti J, Mena M, Unteriner J, Tillotson K, Yin Z, et al. Self-Assessment Feedback Form Improves Quality of Out-of-Hospital CPR. Prehospital Emergency Care 2019;23:66–73. <https://doi.org/10.1080/10903127.2018.1477887>.

[46]

Weston BW, Jasti J, Mena M, Unteriner J, Tillotson K, Yin Z, et al. Self-Assessment Feedback Form Improves Quality of Out-of-Hospital CPR. Prehospital Emergency Care 2019;23:66–73. <https://doi.org/10.1080/10903127.2018.1477887>.

[47]

Kill C, Rupp D, Hartmann H, Wranze E, Müller M, Plöger B. Strukturiertes Team-Feedback bei der Reanimation: Ein Verfahren zur Verbesserung der Versorgungsqualität beim präklinischen Kreislaufstillstand. Notfall Rettungsmed 2016;19:86–91. <https://doi.org/10.1007/s10049-015-0115-8>.

[48]

Bohn A, Weber TP, Wecker S, Harding U, Osada N, Van Aken H, et al. The addition of voice prompts to audiovisual feedback and debriefing does not modify CPR quality or outcomes in out of hospital cardiac arrest – A prospective, randomized trial. Resuscitation 2011;82:257–62. <https://doi.org/10.1016/j.resuscitation.2010.11.006>.

[49]

Taku I. The Effectiveness of high performance CPR for out-of-hospital cardiac arrests: a randomized control trial n.d. <https://upload.umin.ac.jp/cgi-open-bin/ctr_e/ctr_view.cgi?recptno=R000024721> (accessed March 23, 2020).

[50]

Spaite DW, Crawford S, Venuti M, Mason T, Walka P, Smith G, et al. The impact of scenario-based training and real-time technology feedback on CPR quality and survival from out-of-hospital cardiac arrest. Academic Emergency Medicine 2011;18:S4–249. <https://doi.org/10.1111/j.1553-2712.2011.01073.x>.

[51]

Abella BS. The importance of cardiopulmonary resuscitation quality: Current Opinion in Critical Care 2013;19:175–80. <https://doi.org/10.1097/MCC.0b013e328360ac76>.

[52]

Hellevuo H, Sainio M, Tenhunen J, Hoppu S. The quality of manual chest compressions during transport – Can we handle mattress effect with feedback devices. Resuscitation 2012;83:e47. <https://doi.org/10.1016/j.resuscitation.2012.08.119>.

[53]

Hellevuo H, Sainio M, Huhtala H, Olkkola KT, Tenhunen J, Hoppu S. The quality of manual chest compressions during transport – effect of the mattress assessed by dual accelerometers. Acta Anaesthesiologica Scandinavica 2014;58:323–8. <https://doi.org/10.1111/aas.12245>.
